# 30 Reconstruction Deep Partial Thickness Burns with Ovine Forestomach Matrix: Interim Results from Prospective Observational Study

**DOI:** 10.1093/jbcr/iraf019.030

**Published:** 2025-04-01

**Authors:** John Loftus, Michael Young, Nidhi Aravapalli, Nicole Bernal, Ariel Rodgers, Laura Pezzopane, Beth McGuire, Chinaemelum Akpunonu, Patrick Kennedy

**Affiliations:** Ohio State Comprehensive Burn Center; Ohio State University, Wexner Medical Center; Ohio State University; Ohio State University, Wexner Medical Center; Ohio State University, Wexner Medical Center; Ohio State University, Wexner Medical Center; Ohio State University; Ohio State University, Wexner Medical Center; The Ohio State University College of Medicine

## Abstract

**Introduction:**

Traumatic burns vary in depth, size, and severity, presenting significant challenges to effective treatment. Efficient healing, pain control, good cosmesis, and patient satisfaction are critical goals in burn care, which can significantly reduce hospital burden and enhance patient outcomes. This study evaluated the effectiveness of ovine forestomach matrix (OFM) in promoting rapid healing, minimizing pain, and improving cosmesis in deep partial thickness burns.

**Methods:**

Patients were enrolled into an ongoing prospective, single arm observational registry (NCT05243966) at a certified burn center and who received OFM for burn treatment between August 2023 and June 2024. The burns were debrided following hospital standard of care and OFM was applied to augment healing. Outcome measures included post-operative complications, time to healing, pain, and a patient and observer scar assessment.

**Results:**

Fifteen burn patients with a total of 32 burns were enrolled in the study. The average participant age was 46 years. All burns included areas of deep partial-thickness burns, with a mean burn size of 158cm2 ± 271cm2. All patients were treated with a single application of OFM. One patient received a skin graft (STSG) to a portion of the burn, and one patient was lost to follow-up prior to complete healing. The median healing time for the remaining 14 patients was 18±19 days. All patients achieved complete healing with a single OFM application. The average follow-up time was 140 days ± 63 days. There were no wound complications associated with the use of OFM. Pain scores remained minimal throughout the treatment (median; 0/10), while patient scar satisfaction was 5/5.

**Conclusions:**

This prospective observational study demonstrates that OFM facilitates rapid burn healing with minimal pain, good cosmesis and patient satisfaction, and limited need for additional interventions. OFM offers a cost-effective and patient-friendly approach to managing deep partial thickness burns while ensuring optimal outcomes.

**Applicability of Research to Practice:**

The use of OFM for deep partial thickness burns shows promise in clinical practice by facilitating rapid healing, minimizing pain, and improving cosmesis with minimal complications. This single-application approach could streamline burn management, reduce hospital stays, and enhance patient satisfaction, making it a practical, cost-effective solution for burn care.

**Funding for the Study:**

The study was supported by AROA Biosurgery Limited.

